# Manifestation of Susac syndrome during interferon beta-1a and glatiramer acetate treatment for misdiagnosed multiple sclerosis: a case report

**DOI:** 10.1186/s12886-021-02101-3

**Published:** 2021-09-30

**Authors:** Joanna Roskal-Wałek, Michał Biskup, Magdalena Dolecka-Ślusarczyk, Anita Rosołowska, Andrzej Jaroszyński, Dominik Odrobina

**Affiliations:** 1 Clinic of Ophthalmology, Voivodeship Regional Hospital, Kielce, Poland; 2 Collegium Medicum, Jan Kochanowski University, Kielce, Poland; 3 Clinic of Internal Medicine, Voivodeship Regional Hospital, Kielce, Poland; 4 Clinic of Neurology, Voivodeship Regional Hospital, Kielce, Poland; 5 Ophthalmology Clinic Boni Fratres Lodziensis, Łódź, Poland

**Keywords:** Susac syndrome, multiple sclerosis, glatiramer acetate, interferon beta-1a, case report

## Abstract

**Background:**

Susac syndrome (SS) is characterized by the triad of encephalopathy, branch retinal artery occlusion, and sensorineural hearing loss. However, the diagnosis of SS remains difficult because the clinical triad rarely occurs at disease onset, and symptom severity varies. SS symptoms often suggest other diseases, in particular multiple sclerosis (MS), which is more common. Misdiagnosing SS as MS may cause serious complications because MS drugs, such as interferon beta-1a, can worsen the course of SS. This case report confirms previous reports that the use of interferon beta-1a in the course of misdiagnosed MS may lead to exacerbation of SS. Moreover, our case report shows that glatiramer acetate may also exacerbate the course of SS. To the best of our knowledge, this is the first reported case of exacerbation of SS by glatiramer acetate.

**Case presentation:**

We present a case report of a patient with a primary diagnosis of MS who developed symptoms of SS during interferon beta-1a treatment for MS; these symptoms were resolved after the discontinuation of the treatment. Upon initiation of glatiramer acetate treatment, the patient developed the full clinical triad of SS. The diagnosis of MS was excluded, and glatiramer acetate therapy was discontinued. The patient’s neurological state improved only after the use of a combination of corticosteroids, intravenous immunoglobulins, and azathioprine.

**Conclusions:**

The coincidence of SS signs and symptoms with treatment for MS, first with interferon beta-1a and then with glatiramer acetate, suggests that these drugs may influence the course of SS. This case report indicates that treatment with glatiramer acetate may modulate or even exacerbate the course of SS.

## Background

Susac syndrome (SS) is a rare autoimmune disease in which occlusion of the microvessels in the brain, retina and inner ear leads to a characteristic triad of clinical symptoms: encephalopathy, visual impairment related to branch retinal artery occlusion (BRAO) and hearing loss, respectively [1–3]. SS is also characterized by a neuroimaging triad consisting of white matter lesions, grey matter lesions, and leptomeningeal enhancement on magnetic resonance imaging (MRI) [2].

SS is rare, with an annual incidence of 0.024 per 100,000 people (95 % CI 0.010–0.047) [4]. SS affects women more often than men, and typically occurs between the ages of 20 and 40 years [3].

A definitive diagnosis of SS is made when a clinical or neuroimaging triad is present. Patients do not usually present with a complete clinical or neuroimaging triad initially, which makes diagnosis difficult [2]. Moreover, the symptoms may lead to suspicion of other, more frequently recognized, diseases, such as multiple sclerosis (MS) [1].

Misdiagnosing SS as MS may not only leads to the delayed diagnosis that can worsen the prognosis but may also cause serious complications because MS drugs can worsen the course of SS [5–7]. This case report confirms previous reports that the use of interferon beta-1a in the course of misdiagnosed MS may lead to exacerbation of SS [5, 6]. Moreover, our case report shows that glatiramer acetate may also exacerbate the course of SS. To the best of our knowledge, this is the first reported case of exacerbation of SS by glatiramer acetate.

## Case presentation

**Fig. 1:**
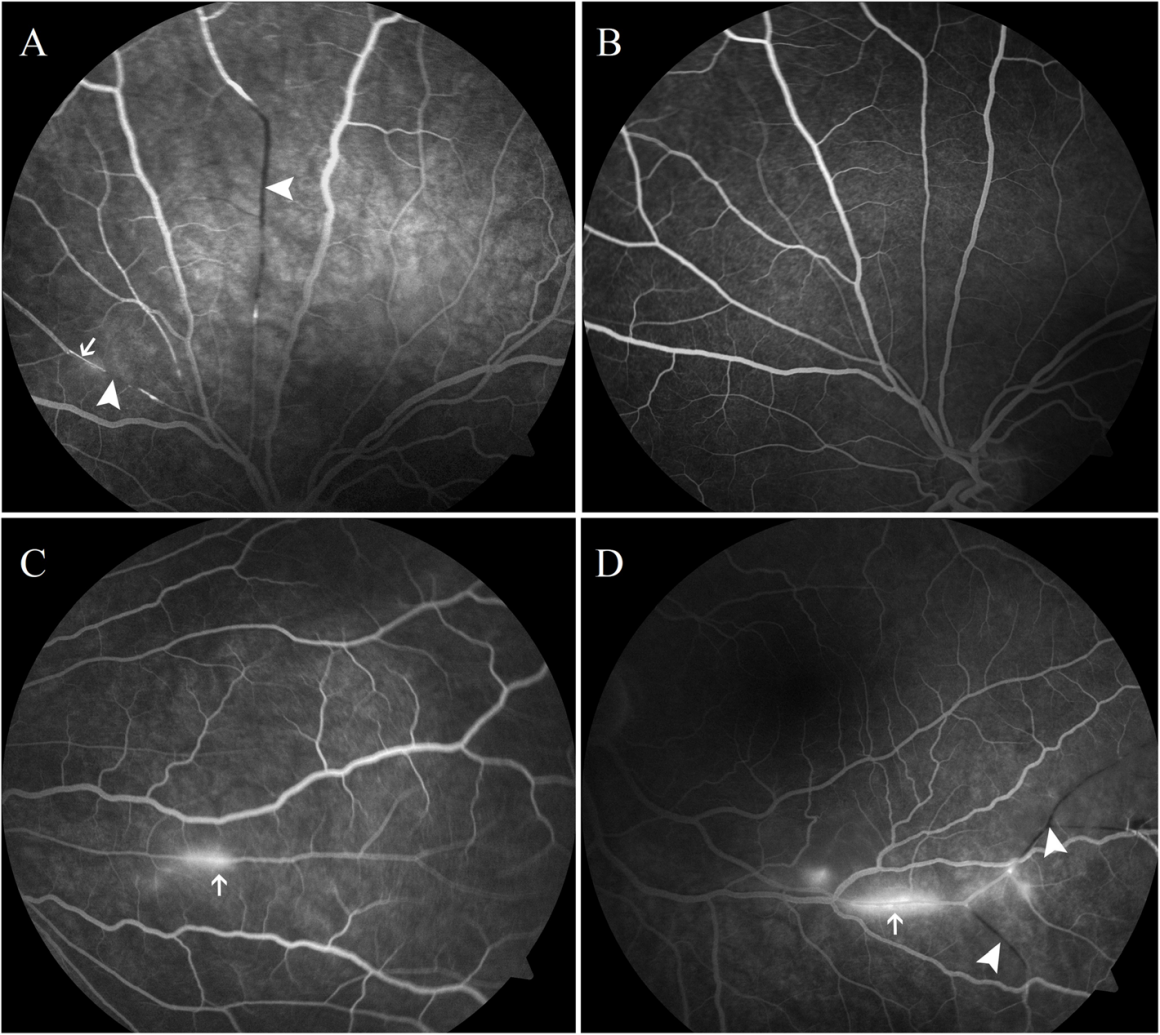
**A** First fluorescein angiography of the left eye: Late phase shows segmental arteriolar wall hyperfluorescence (arrows) and branch retinal artery occlusion (arrowheads). **B** Second fluorescein angiography of the left eye performed two weeks later shows reperfusion of the previously occluded retinal arterioles and resolution of the arteriolar wall hyperfluorescence. **C** Following fluorescein angiography of the right eye shows arteriolar wall hyperfluorescence (arrows) in a peripheral branch. **D** Following fluorescein angiography of the left eye shows a new branch retinal artery occlusion (arrowheads) and arteriolar wall hyperfluorescence (arrows)

A 20-year-old woman receiving interferon beta-1a for MS reported a visual field defect in the lower temporal quadrant of the left eye. Examination revealed a normal visual acuity of 20/20 in both eyes. Intraocular pressure was 15 mmHg in the right eye and 17 mmHg in the left eye. Anterior segment examinations were normal in both eyes. Pupils were equal, round and reactive to light with no relative afferent pupillary defect. Fundus examination of the left eye showed ischemic retinal whitening in the supra-nasal area, fluorescein angiography (FA) revealed BRAOs and subtle, segmental arteriolar wall hyperfluorescence (AWH) at the site of BRAO in the late phase (Fig. 1 A). Fundus examination and FA of the right eye were normal. Retrobulbar optic neuritis due to MS was ruled out because the infra-temporal visual field defect reported by the patient corresponded to the area of the ischemic retina due to supra-nasal BRAO. Moreover, the patient did not have reduced visual acuity nor colour vision disturbances, and did not report any pain concomitant to eye movements which is characteristic for retrobulbar optic neuritis in the course of MS. Interferon beta-1a treatment was discontinued after 7 weeks because of its possible prothrombotic effect. A repeat FA performed two weeks later showed reperfusion of the occluded arterioles and resolution of the AWH (Fig. 1 B).

The patient had increased thromboembolic risk due to hormonal contraception and cigarette smoking. A full laboratory work-up was done, including tests for connective tissue disease, vasculitis, borrelia, syphilis, human immunodeficiency virus (HIV), herpes simplex virus (HSV), cytomegalovirus (CMV), and factor V Leiden mutations. The results of laboratory studies (borderline lupus anticoagulant, leukopenia, decreased platelet count, slightly elevated D-dimer concentration, and prolonged activated partial thromboplastin time) were suggestive of antiphospholipid syndrome or lupus; however, further biochemical tests excluded these causes. Performed again with an interval of 12 weeks, anti-cardiolipin antibodies in the IgG or IgM class, lupus anticoagulant and antibodies against β2-glycoprotein were negative. Moreover, IgM and IgG antibodies against CMV were detected. Transthoracic echocardiography and carotid artery ultrasonography were unremarkable.

**Fig. 2:**
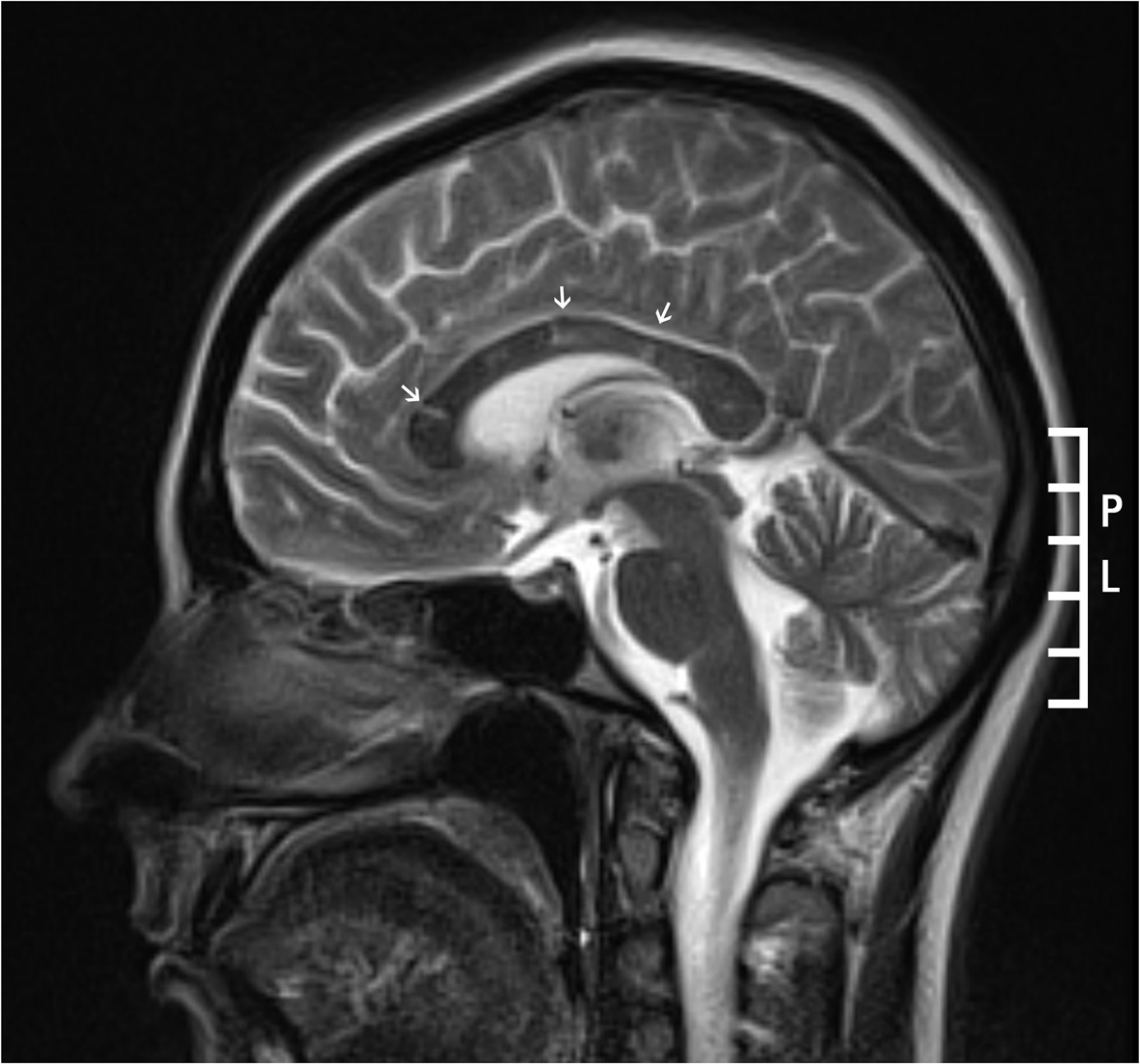
Brain MRI (T2-weighted sequence, sagittal view) shows lesions (arrows) in the trunk and the genu of the corpus callosum consistent with the diagnosis of Susac syndrome. Some of the lesions have typical spoke-like appearance

Treatment with glatiramer acetate was started 3 weeks after discontinuation of interferon beta-1a. After 2 weeks of glatiramer acetate therapy, we observed neurological worsening with fever, headache, impaired consciousness, left-sided weakness, and lower limb ataxia. Lumbar puncture revealed only a mild elevation of cerebrospinal fluid protein, no oligoclonal bands, and a negative encephalitis panel; meningitis was therefore ruled out. The brain MRI showed diffuse and limited hyperintense changes in fluid-attenuated inversion recovery (FLAIR) and T2 sequences located periventricularly in the subcortical white matter, mainly in the frontal and parietal lobes, in the pons, in the basal ganglia and in the corpus callosum. MRI revealed also post contrast leptomeningeal enhancement (Fig. 2). Repeated FA showed new BRAOs and AWH in both eyes (Fig. 1 C, D). Moreover, the patient reported hearing loss; however, pure tone audiometry was inconclusive because of the patient’s worsening condition. We also noted livedo reticularis and a maculopapular rash. SS was diagnosed based on these new findings. The diagnosis of MS was excluded, and glatiramer acetate therapy was discontinued. Treatment was started with Methylprednisolone (0.5–1.0 g) which was administered for 5 days; the total used dose was 3.0 g. Despite slight initial improvement, neurological deterioration occurred after 7 days when the steroids dose was reduced to 50 mg prednisone per day (a total used dose of 350 mg of prednisone). Neither plasma exchange (four courses) nor azathioprine improved the patient’s neurological condition. The patient’s neurological state improved only after use of a combination of corticosteroids, intravenous immunoglobulins, and azathioprine; visual acuity was 20/20 in both eyes, however in the fundus examination, BRAO’s in the peripheral retinal artery branches were still present in the right and left eye.

## Discussion and conclusions

The clinical manifestation of SS is highly variable. The complete triad is present in only 13 % of patients at disease onset. Any one of the triad components may initially be the only symptom, which can lead to a delayed or wrong diagnosis. Full manifestation of SS is observed within several months, and in some cases complete manifestation of SS lasted for more than two years [8]. In our case, the patient did not initially present the full clinical triad, which led to a misdiagnosis of MS with the exacerbation of SS symptoms, first during interferon beta-1a treatment, and then during glatiramer acetate treatment.

Significant progress has recently been made in understanding SS, making it easier to differentiate from MS and other entities [1–3]. Egan reported two pathognomonic imaging findings of SS that can be observed on MRI and FA [2]. On MRI, SS involves the corpus callosum more frequently than MS. Lesions in the central part of the corpus callosum caused by infarction of tiny arterioles, which present on MRI as “snowballs” or “spokes”, are considered pathognomonic of SS. By contrast, in MS, changes are observed on the undersurface of the corpus callosum and in the callososeptal interface [1, 2]. In patients with SS, BRAO and AWH are observed on FA. AWH is the result of a characteristic leakage and indicates damage to the tight junctions and the integrity of the vessel wall [1, 2]. AWH at the site of BRAO is nonspecific; however, AWH observed away from the site of BRAO is pathognomonic of SS [2].

According to previously published criteria, patients without a complete clinical triad but with central callosal lesions on MRI or AWH on FA would be diagnosed with probable SS [9]. The new criteria for the diagnosis of SS proposed by Egan recognize central callosal lesions on MRI or AWH on FA distant from BRAO as diagnostic for SS, even in the absence of a full clinical or neuroimaging triad. Identifying the pathognomonic signs of SS may speed up the diagnosis of SS, which is crucial for prognosis [2]. A prompt and correct diagnosis of SS can prevent a patient from receiving inappropriate treatment.

Misdiagnosis of SS as MS can cause serious complications. Drugs such as interferon beta-1a or natalizumab have been reported to worsen the course of SS [5–7]. However, there are reports that natalizumab may be effective for the treatment of SS [3]. Exacerbations of SS are assumed to result from changes to the immune system caused by these drugs [1].

As in the case described by Laird et al. [6], our patient developed BRAO and AWH during interferon beta-1a treatment, and these symptoms resolved shortly after discontinuation of the drug, suggesting that interferon beta-1a may have contributed to the development of this ocular manifestation of SS. The observed changes could also be atypical signs of retinopathy in the course of interferon beta-1a treatment. The visual impairment caused by BRAO required verification of the diagnosis of MS. The occurrence of retinopathy in a patient with MS is one of the major red flags and points to a non-MS diagnosis, SS for example [10].

In the differential diagnostics of retinal artery occlusion in young persons, haematologic disorders, factor V Leiden, protein C and S and anti-thrombin deficiencies, prothrombin gene mutations, sickle cell anaemia, migraine secondary to vasospasm, vasculitis, systemic lupus, antiphospholipid syndrome, or use of oral contraceptives, as well as valve disorders, should also be taken into consideration [11, 12].

In our case, the positive lupus anticoagulant test, together with other laboratory abnormalities, was suggestive of antiphospholipid syndrome or systemic lupus, however, further biochemical tests excluded these causes. Anticardiolipin antibodies and lupus anticoagulant have also been detected in patients with SS, but whether they are pathogenic is uncertain [13].

During treatment with glatiramer acetate, BRAO and AWH were again detected on FA; this time, AWH was localized away from the site of BRAO, which is pathognomonic for SS. Our patient’s neurological condition deteriorated after the use of glatiramer acetate, and MRI showed changes in the central part of the corpus callosum, indicating SS. There was also hearing impairment. To our knowledge, we are the first to report that glatiramer acetate may affect the course of SS.

We cannot rule out the possibility that this was the natural course of SS in this patient. Nevertheless, the coincidence of SS signs and symptoms with treatment for MS, first with interferon beta-1a and then with glatiramer acetate, suggests that these drugs may influenced the course of SS.

This is an interesting report both due to the proposed mechanisms of action of glatiramer acetate and the recently described new model of SS pathogenesis [3, 14]. The mechanism of action of glatiramer acetate is still not entirely clear — although most attention is focused on the effects of glatiramer acetate on CD4 T cells, it also greatly enhances the CD8 T cell response [14]. Gross et al. demonstrated that cytotoxic CD8 T cells mediated the vascular injury to the central nervous system in SS and reported cytotoxic T cell-dependent endotheliopathy against an unidentified antigen to be the major pathogenic process [3].

Additionally, both interferon beta-1a and glatiramer acetate modulate and interfere with the immune response, potentially increasing susceptibility to infection [15]. One hypothesis concerning the pathophysiology of the disease is a parainfectious mechanism involving the presentation of viral antigen on the endothelium after viral infection [3]. In the study by Wilf-Yarkoni et al., results of analyses for CMV infection were available for four of seven patients with SS; three of these patients had anti-CMV IgM antibodies. Wilf-Yarkoni et al. therefore suggest that an inflammatory mechanism may contribute to the development of SS [16]. Our patient also had positive IgG and IgM antibodies to CMV.

Based on their research, Gross et al. point out that, although it also induces CD8 + T cells, CMV is not a driving force in SS [3]. Nevertheless, the influence of an infectious trigger in the pathogenesis of SS requires further investigation. Recently, Venditti et al. reported a case of SS after COVID-19 [17].

In conclusion, distinguishing SS from MS is a diagnostic challenge. The presented case is an example of how difficult it is to correctly diagnose SS in a patient with primary misdiagnosed MS who additionally has risk factors for BRAO and a positive CMV antibody. Misdiagnosis can lead to serious problems caused by inappropriate therapy. This case report suggests that treatment with glatiramer acetate may modulate or even exacerbate the course of SS. Further research is required to confirm this hypothesis.

Our observations also raise awareness of the importance of the early and correct diagnosis of SS. This case also highlights the importance of interdisciplinary collaboration for the correct diagnosis of SS. Ophthalmological evaluation of patients with MS is essential for differential diagnosis and, in some cases, may be key to achieving a correct diagnosis of SS, which translates directly into improved prognosis.

## Data Availability

The datasets used and analysed during the current study are available from the corresponding author on reasonable request.

## Abbreviations

SS: Susac syndrome
MS: multiple sclerosis
BRAO: branch retinal artery occlusion
MRI: magnetic resonance imaging
AWH: arteriolar wall hyperfluorescence
FA: fluorescein angiography
HIV: human immunodeficiency virus
HSV: herpes simplex virus
CMV: cytomegalovirus
